# A White doctor recalls mental hospital practice in apartheid South Africa

**DOI:** 10.1192/bji.2021.36

**Published:** 2021-11

**Authors:** Harold Behr

**Affiliations:** MBBCh, DPM, FRCPsych, Consultant in Child Psychiatry (retired), formerly at Central Middlesex Hospital, London, UK. Email harold.behr@ntlworld.com

**Keywords:** Stigma and discrimination, transcultural psychiatry, electroconvulsive therapy, history of psychiatry, psychotic disorders

## Abstract

The article offers a personal memoir of a psychiatric registrar's impressions of daily life while working in a South African mental hospital during the apartheid era in the late 1960s. From the perspective of a trainee psychiatrist, he recalls admission procedures, ward management, patient assessment, and medical and nursing care, including electroconvulsive therapy administration, at that time.

More than 50 years ago I was a registrar in psychiatry at Witwatersrand University Medical School in Johannesburg, South Africa. As part of the training I worked for 6 months at Sterkfontein Mental Hospital near the town of Krugersdorp and it was there that I recorded the following impressions, which I hope will be of interest to readers as a glimpse into the past and an awareness of ground still to be covered. The year is 1968.

## Admission

Every few days a police van draws up outside the Admission ward in the ‘Non-European’ section of this large mental hospital, and disgorges its passengers. The people who climb uncertainly out of the van have all been committed to the hospital under the Mental Disorders Act. Some are pitifully neglected, malnourished and filthy. Most of them file unobtrusively into the ward, but one or two parade the bizarre behaviour that has brought them to their latest destination and have to be firmly steered into the custody of waiting nurses.

A common story is that they have been picked up as vagrants or that they have been involved in some violent incident in one of the sprawling townships that lie on the outskirts of the White residential areas. In a society where eccentric behaviour is more likely to attract a police van than an ambulance, these disturbers are moved to a police station, which then serves as a convenient half-way house out of the community.

They are kept in cells until they can be examined by a district surgeon, who decides whether or not they should be committed. The examination consists of a brief interview to establish whether there are legally acceptable criteria for the committal and to conduct a perfunctory physical check to rule out any somatic illness which might require urgent medical attention. The condition of some of the individuals who arrive at the hospital is sometimes strangely at odds with the routine health clearance entered on their medical certificates.

Most of the new arrivals are too wrapped up in their illness to know what is happening to them. Some think that they have simply been transferred to another jail. Inside the ward they are subjected to a handing-over ritual in which the police officer formally divests himself of responsibility for their care and transfers it to the nurse in charge of the ward. To bring this into effect, the soon-to-be-patients are ordered to strip naked in the presence of a doctor, who then peers at them somewhat in the manner of a slave-trader inspecting merchandise, looking at them from all angles, swivelling them around and scrutinising them for signs of injuries that might have a medico-legal comeback. Wrists and ankles are particularly looked at for signs of chafing, because some of these people will have been forcibly restrained at some time in the stormy prelude to their admission.

All injuries are recorded in detail and a receipt is handed to the accompanying policeman containing a brief comment on the patient's condition. The legal papers required for admission are carefully studied to ensure that everything is in order before the patients can be accepted into the hospital.

## On the ward

From the admissions office the patients are led out to become part of the ward population, numbering about 100. The first rudimentary steps in the patching-up operation that follows the admission are taken when the patient is bathed and fitted out with hospital clothes, consisting, in the case of men, of a pair of baggy shorts and a jersey with the ward's number embroidered on it.

Life in the ward is a monotonous routine. Most of the patients are confined to a large central yard, where they roam around aimlessly or lie on the grass until they are summoned to meals, bed or interviews with the staff, or to receive medication. The more obviously disturbed members of this throng provide a grotesque spectacle as they walk to and fro manneristically or assume postures in a world of their own. Doctors, identified by their white coats, are sometimes cheerfully hailed as they thread their way along the rim of the yard.

The entrance to the yard is always locked. The only patients allowed into the hospital grounds are those organised into work parties to tend the gardens, which are beautifully kept. The wards, too, are spotlessly clean. The atmosphere is usually thick with the smell of polish and disinfectant.

The food, dished out in ample dollops, is nutritious. Patients sit at long wooden benches to eat and make do with crude utensils. Beds in dormitories are arranged in long rows to facilitate observation, while toilets and bathrooms, no doubt with the same objective in mind, lack privacy. For those patients who might have seen better days, such conditions are a grim reminder of their ordained role in the apartheid society.

## The staff

In line with government policy, Black members of staff are consigned to permanent subordination in the hierarchy. In the face of this iniquitous system it is ironic that many of the staff, both Black and White, are people of great integrity. They show a touching concern for the patients in their care and pride themselves on the efficiency with which their wards are run.

In keeping with the authoritarian culture, psychiatrists are ensconced at the top of the professional pyramid and psychiatry is largely a male preserve. Wards are partitioned into ‘male’ and ‘female’ and housed in separate buildings. This mirrors the gender composition of the attached nursing staff. Case conferences are routinely held at which opinions are canvassed from any professional who happens to be present, but important decisions about the patients’ therapy and management remain the preserve of the senior doctor. The role of the clinical psychologist is more or less restricted to the administration of psychometric tests, usually to patients under observation pending a criminal charge. Psychotherapy is unheard of, even at a basic level of structured, supportive interviews.

Charge nurses bear the brunt of the responsibility for the patients’ day-to-day care. They administer emergency medication, often with carte blanche from the doctors or advice given over the phone, and they are heavily relied on by the doctors for their observations of the patients’ progress. Some of the veteran nurses have strong views on treatment and do not hesitate to make their recommendations known to the trainee psychiatrist who is nominally in charge of *ad hoc* prescribing. Great faith is placed in electroconvulsive therapy. ‘Give him a bit of shock, doctor,’ is an often heard piece of advice.

## Patient assessment

Much of the doctor's time is spent making periodic assessments of the patients’ mental state in accordance with a strictly defined manual. Assessments are carried out with decreasing frequency as the duration of the stay in hospital lengthens. Newly admitted patients are seen weekly for the first month. Thereafter, interviews are tailed off to one a month for the first year's stay, then every 3 months, ending up with long-stay patients being seen at 6-monthly intervals.

These interviews are arranged with military efficiency. The charge nurse informs the doctor when a batch of patients is ready to be seen. These patients are then gathered together by the ward staff and assembled on benches outside the office that serves as an interview room. The doctor sits at a desk on which the case notes of the patients due to be seen (the ‘periodicals’) are stacked and the charge nurse then ushers the patients in one by one to be seated beside the doctor.

Since many of the patients only speak a poor English or Afrikaans and few of the doctors speak an African language, a Black nurse, standing deferentially in the background, interprets for the doctor. Questions are put in only the simplest and most concrete of terms, which means that any nuanced exploration of the patient's own concerns, family relationships or economic circumstances falls by the wayside. The question–answer routine is confined to a phenomenological enquiry into the patient's current mental state. Typically, is the patient orientated as to time, place and person? Do they harbour beliefs that might be regarded as delusional? Are they experiencing hallucinations?

Some patients are unable to respond verbally, others have to be helped or even carried into the interview room, and the interview takes on the form of an inspection rather than an exchange. One category of patients, those who are helpless to the point of having to be fed and changed, are familiarly referred to as the ‘wet-and-dirties’. For these patients diagnostic niceties have long since given way to the main consideration of nursing care.

Many of the patients in the long-stay wards (the so-called ‘chronic’ wards) have been there for decades and their case notes are ancient manuscripts providing a quaint anthology of psychiatric styles sometimes more suited to a museum display cabinet than a clinical filing system. After a detailed initial entry, the reports tend to become more telegrammatic, resulting in overworked phrases such as ‘dull and apathetic’, ‘withdrawn’, ‘emotionally blunt’, ‘continues to be hallucinated’. A tendency to violence or aggressiveness is sometimes noted, though without much reference to the context.

## Electroconvulsive therapy

Electroconvulsive (‘shock’) therapy (ECT) is often prescribed and it is quite usual for forty or fifty patients to be receiving this treatment on any given day. A dormitory ward is converted into a temporary treatment room by laying down rolls of bedding along the length of the ward. Those scheduled for the treatment are shepherded into an adjoining bathroom, from where they are led out one by one to receive the treatment.

Occasionally a patient balks while being led along and tries to make a run for it, at which a scuffle ensues as several nurses struggle to contain the patient. Once the patient has been laid down and is momentarily still, the headpieces are clamped to the temples and the doctor switches on the current, plunging the patient into instant unconsciousness. Several attendants grasp the limbs firmly during the convulsion to minimise the risk of injury. The preliminary administration of a general anaesthetic to relax the musculature, normally considered a humane procedure when administering ECT, is deemed unnecessary.

The end of the seizure is usually signalled by a deep sigh, at which point the patient is deftly turned over on to their side and covered with a blanket. By this time, the next patient is already lying in position and the procedure can be repeated, the headpieces simply being transferred from one patient to the other. The ECT machine, mounted on a trolley, is wheeled down the centre aisle, leaving in its wake two rows of patients in varying degrees of stupor – some already climbing groggily to their feet, others snoring in the deep sleep that sometimes follows a seizure.

## Looking back

My experiences during my placement at Sterkfontein Hospital over half a century ago left me with a sense of unstinting admiration for the hospital staff, whose devotion to their patients was beyond reproach. In particular, I recall the genuine warmth and compassion expressed by White staff towards their Black charges, which transcended their known ideological bias towards apartheid.

At the same time I was appalled by the differences between mental and physical healthcare in the community and by the iniquitous segregation of facilities imposed on the hospital by the apartheid system. I had already decided to leave South Africa after completing my training, and my time at Sterkfontein only strengthened my resolve to do so. In retrospect, my subsequent choice of subspecialties (child psychiatry and psychotherapy) was probably in part determined by the traumatic experiences that had an impact on me during that period of my training.

